# Impact of delays to incubation and storage temperature on blood culture results: a multi-centre study

**DOI:** 10.1186/s12879-021-05872-8

**Published:** 2021-02-12

**Authors:** Clare L. Ling, Tamalee Roberts, Sona Soeng, Tomas-Paul Cusack, David A. B. Dance, Sue J. Lee, Thomas A. N. Reed, Pattaraporn Hinfonthong, Somsavanh Sihalath, Amphone Sengduangphachanh, Wanitda Watthanaworawit, Tri Wangrangsimakul, Paul N. Newton, Francois H. Nosten, Paul Turner, Elizabeth A. Ashley

**Affiliations:** 1 Shoklo Malaria Research Unit, Mahidol-Oxford Tropical Medicine Research Unit, Faculty of Tropical Medicine, Mahidol University, Mae Sot, Thailand; 2 Centre for Tropical Medicine and Global Health, Nuffield Department of Medicine, University of Oxford, Oxford, United Kingdom; 3 Lao-Oxford-Mahosot Hospital-Wellcome Trust Research Unit, Microbiology Laboratory, Mahosot Hospital, Vientiane, Lao People’s Democratic Republic; 4 Cambodia Oxford Medical Research Unit, Angkor Hospital for Children, Siem Reap, Cambodia; 5 Present address: Department of Infection, University Hospital Southampton, Southampton, UK; 6 Faculty of Infectious and Tropical Diseases, London School of Hygiene and Tropical Medicine, London, UK; 7 Mahidol-Oxford Tropical Medicine Research Unit, Faculty of Tropical Medicine, Mahidol University, Bangkok, Thailand; 8 Present address: London North West University Healthcare NHS Trust, Northwick Park Hospital, London, UK; 9 Myanmar Oxford Clinical Research Unit, Yangon, Myanmar

**Keywords:** Blood cultures, Delays, LMIC, Diagnostics, Microbiology

## Abstract

**Background:**

Blood cultures are one of the most important tests performed by microbiology laboratories. Many hospitals, particularly in low and middle-income countries, lack either microbiology services or staff to provide 24 h services resulting in delays to blood culture incubation. There is insufficient guidance on how to transport/store blood cultures if delays before incubation are unavoidable, particularly if ambient temperatures are high. This study set out to address this knowledge gap.

**Methods:**

In three South East Asian countries, four different blood culture systems (two manual and two automated) were used to test blood cultures spiked with five common bacterial pathogens. Prior to incubation the spiked blood culture bottles were stored at different temperatures (25 °C, in a cool-box at ambient temperature, or at 40 °C) for different lengths of time (0 h, 6 h, 12 h or 24 h). The impacts of these different storage conditions on positive blood culture yield and on time to positivity were examined.

**Results:**

There was no significant loss in yield when blood cultures were stored < 24 h at 25 °C, however, storage for 24 h at 40 °C decreased yields and longer storage times increased times to detection.

**Conclusion:**

Blood cultures should be incubated with minimal delay to maximize pathogen recovery and timely result reporting, however, this study provides some reassurance that unavoidable delays can be managed to minimize negative impacts. If delays to incubation ≥ 12 h are unavoidable, transportation at a temperature not exceeding 25 °C, and blind sub-cultures prior to incubation should be considered.

**Supplementary Information:**

The online version contains supplementary material available at 10.1186/s12879-021-05872-8.

## Background

Blood cultures (BCs) are one of the most important tests performed by microbiology laboratories. Usually taken from the most unwell patients, they may reveal the likely source of infection and guide antibiotic therapy. Moreover, they can provide reliable surveillance data for informing national antibiotic treatment guidelines. The Global Antimicrobial Resistance Surveillance System (GLASS) of the World Health Organization identifies BCs as key for antimicrobial resistance surveillance in all Member States and many low and middle-income countries (LMICs) are increasing their laboratory capacity to meet this target [1].

Several initiatives have been evaluated to reduce the time to identify and test antimicrobial susceptibility of pathogens causing bacteremia with the joint goals of improving patient management and antimicrobial stewardship, for example around-the-clock processing and direct antimicrobial susceptibility testing from positive BC bottles [2, 3]. However, all these attempts can be undermined by transportation delays or other factors leading to delays in incubating BCs. Such delays occur when laboratories do not operate a 24 h service, which is common in LMICs or when BCs are referred from other hospitals to a centralized laboratory, also common in LMICs and increasingly in other settings due to consolidation of pathology services [4, 5]. Delayed incubation leads not only to delayed pathogen identification but may also affect the diagnostic yield of BCs [6, 7]. In addition, in some LMICs BCs are likely to be stored at tropical ambient temperatures that can exceed recommended incubation temperatures.

This study investigated the effects of storage for different times at different temperatures on the isolation of some important bacteria frequently grown in BCs. To increase the generalizability of the results, the study was performed in three South East Asian countries using two different manual and two different automated BC systems. Pediatric bacteremia was simulated, with effects on yield and time to positivity (TTP) explored.

## Methods

### Sites

The study took place in laboratories at: the Cambodia Oxford Medical Research Unit (COMRU), Siem Reap, Cambodia; the Shoklo Malaria Research Unit (SMRU), Tak Province, Thailand; and the Lao-Oxford-Mahosot Hospital-Wellcome Trust Research Unit (LOMWRU), Vientiane, Lao PDR.

### Blood cultures

Manual BC systems were used at COMRU and LOMWRU, and automated BC systems were used at LOMWRU and SMRU. All sites used pediatric aerobic BC bottles. At COMRU, BC bottles contained 25 ml of Brain Heart Infusion broth plus 0.025% sodium polyanethol sulfonate (SPS); at LOMWRU, BC bottles for their manual method contained 20 ml tryptic hydrolysate of casesin and soy peptone broth (TSB) with 0.05% SPS while PEDS Plus™/F bottles (Becton Dickinson, New Jersey, USA) were used with the automated BD BACTEC™ system and at SMRU, PF Plus bottles were used with the automated BacT/ALERT® system.

BC bottles were inoculated with 2 ml fresh untreated human blood intended to contain approximately 3 CFU/ml of one of five bacterial strains: *Escherichia coli* (ATCC 25922), *Staphylococcus aureus* (ATCC 25923), *Streptococcus pneumoniae* (ATCC 49619), *Streptococcus agalactiae* (ATCC 12386), or *Haemophilus influenzae* (ATCC 10211). This was achieved by preparing suspensions of each control strain (using less than 24 h old bacterial cultures on solid media) in normal saline equivalent to a 1 McFarland standard (approximately 3 × 10^8^ CFU/ml), further diluting them to approximately 30 CFU/ml with normal saline, and then making a final 1 in 10 dilution with donor blood to obtain a final concentration of approximately 3 CFU/ml. The Miles and Misra method was used to check actual concentrations [8]. The untreated blood was obtained from 13 healthy human volunteers (four each at SMRU and LOMWRU and five at COMRU). All volunteers were > 18 years of age, were not pregnant, had not previously been treated for anemia, and had not knowingly received antibiotics in the previous month. To avoid autoagglutination, volunteer blood was used immediately after collection, with time to bottle inoculation taking < 30 min.

Inoculated bottles were stored at different temperatures and times, with five replicates for each organism/storage condition combination, except LOMWRU where BACTEC bottles were tested in triplicate due to machine capacity (Table 1). Cool-boxes (double-skinned metal boxes) without icepacks, were kept in the shade at ambient temperatures at each site with their internal temperature monitored continuously with TinyTag Transit 2 (Gemini Data Loggers, UK) or MicroLite (Fourtec – Fourier Technologies Ltd., Australia) data loggers.

**Table 1:** Storage conditions and number replicates for blood culture bottles spiked with five different organisms

Storage condition	Per organism					Total
	COMRU^a^	LOMWRU^a^	SMRU^b^	LOMWRU^b^	Total	
No storage	×5	×5	× 5	×3	×18	90
6 h 25 °C	× 5	× 5	× 5	× 3	× 18	90
6 h cool-box	× 5	× 5	× 5	× 3	× 18	90
6 h 40 °C	× 5	× 5	× 5	× 3	× 18	90
12 h 25 °C	× 5	× 5	× 5	× 3	× 18	90
12 h cool-box	× 5	× 5	× 5	× 3	× 18	90
12 h 40 °C	× 5	× 5	× 5	× 3	× 18	90
24 h 25 °C	× 5	× 5	× 5	× 3	× 18	90
24 h cool-box	× 5	× 5	× 5	× 3	× 18	90
24 h 40 °C	× 5	× 5	× 5	× 3	× 18	90
Total	50	50	50	30	180	900

^a^ = manual system; ^b^ = automated system (BacT/ALERT® for SMRU, BD BACTEC™ for LOMWRU)

For BacT/ALERT® PF Plus bottles the color of the bottle sensor, which changes from green to yellow to indicate growth, was recorded pre-incubation. The bottles in the automated systems were incubated until they flagged positive or for at least 5 days. Sub-cultures were performed on bottles that flagged positive to confirm the identification of any viable bacteria and to rule out contamination. For manual BC systems, the bottles were inspected for visible growth and sub-cultured every 24 h until a positive sub-culture was obtained or for a maximum of 7 days.

### Statistical analysis

The percentage of positive cultures detected was summarized by storage condition. Pairwise differences across groups were compared using Fisher’s exact test. The sample size of 90 bottles for each storage condition enabled the detection of a difference in the proportion of culture growth from 89 to 73%, assuming an alpha of 0.05 and 80% power. As a secondary outcome, median TTP (p25, p75) from inoculation was summarized for each storage condition and compared using the Wilcoxon rank-sum test and a test-for-trend across groups. *P* values < 0.05 were considered statistically significant. Analyses were performed using STATA version 15.1 (StataCorp LLC, USA).

## Results

### Inoculum

Using the Miles and Misra method the bottles were confirmed to have been inoculated with blood containing 1 to 10 CFU/ml (median 2.9 CFU/ml) of bacteria, resulting in 2 to 20 CFU/bottle. For *E. coli*, *H. influenzae*, *S. aureus*, *S. agalactiae* and *S. pneumoniae* the median CFU/ml were 2.3 (range 2.0–3.0), 4.5 (range 3.2–7.0), 4.1 (range 2.3–7.0), 2.2 (range 1.7–2.9) and 1.7 (range 1.0–10.0), respectively.

### Sub-culture results

For each organism (*E. coli, H. influenzae, S. agalactiae, S. aureus* and *S. pneumoniae*) 180 bottles were inoculated of which 80.6, 95.0, 90.0, 93.3 and 66.1% were positive respectively. The expected organism was obtained from all positive bottles when they were sub-cultured except for one bottle inoculated with *S. pneumoniae* but grew a *Staphylococcus* species; this bottle was regarded as contaminated and excluded from further analsysis. In addition, at SMRU, 19 bottles inoculated with *S. pneumoniae* flagged positive in the Bact/Alert system, but failed to grow an organism when sub-cultured. These 19 ‘positive’ bottles showed exponential growth on their charts, BC bottle media showed evidence of browning, Gram positive cocci were seen on Gram staining and their median time from flagging positive to sub-culture (13.73 h, IQR 10.05–14.65) was significantly longer than the median time taken to sub-culture the 23 bottles at SMRU inoculated with *S. pneumoniae* that did grow (7.57 h, IQR 0.43–12.07, *P* < 0.001). It was concluded that failure of sub-culture for these 19 bottles was due to autolysis and to avoid bias due to sub-culture delay for this organism, they were included in the analysis as positive bottles.

### Positive blood culture yield

The numbers of positive BC bottles obtained for different storage conditions are shown in Table 2.

**Table 2:** Overall number of positive blood cultures obtained for different storage conditions, compared with no delay; and number of BacT/ALERT® bottle sensors that indicated growth prior to incubation

Time (h)	Temp (°C)	Bottles (n)	Positive (n)^**a**^	Positive (%)	***P*** value^c^	Sensor positive^d^
0	NA	90	80	88.9	NA	0/25
6	25 °C	90	85	94.4	0.281	0/25
6	Cool-box	90	81	90.0	1.000	0/25
6	40 °C	90	74	82.2	0.289	0/25
12	25 °C	90	76	84.4	0.511	0/25
12	Cool-box	89^b^	80	89.9	1.000	0/25
12	40 °C	90	77	85.6	0.656	15/25
24	25 °C	90	82	91.1	0.805	20/25
24	Cool-box	90	71	78.9	0.104	20/25
24	40 °C	90	59	65.6	< 0.001	20/25
6	All	270	240	88.9	1.000	0/75
12	All	269^b^	233	86.6	0.787	15/75
24	All	270	212	78.5	0.030	60/75
All	25 °C	270	243	90.0	0.841	20/75
All	Cool-box	269^b^	232	86.2	0.694	20/75
All	40 °C	270	210	77.8	0.021	35/75

^a^For automated systems this is the number of bottles that flagged positive, and for manual systems this is the number of bottles that were positive on sub-culture; ^b^One bottle excluded due to contamination; ^c^Compared with no delay; ^d^Sensor positive if BacT/ALERT® bottle sensor indicated growth prior to incubation. *NA* Not applicable

When combining results for all organisms, no significant differences in positive bottle yields compared with no delay were detected when bottles were stored for 6 h, 12 h, at 25 °C or in a cool-box. The median (IQR; range) temperature inside the cool-boxes during the study was 27.0 °C (26.0–27.9, 19.8–31.0).

Significantly lower yields compared with no delay were detected when bottles were stored for 24 h or at 40 °C; and the lowest yield was obtained for bottles stored for 24 h at 40 °C. Storage for 24 h at 40 °C gave the lowest positive yield for both manual and automated systems [35/50 (70.0%) and 24/40 (60.0%), respectively].

When stratified by organism, no significant differences for any storage condition compared with no delay were detected for *E. coli*, *H. influenzae,* and *S. aureus*. For *S. agalactiae,* fewer bottles were positive when stored for 24 h at 40 °C (66.7%) and 24 h in a cool-box (55.5%) compared to no storage at all (100%, *P* = 0.019 and 0.003 respectively). For *S. pneumoniae,* storage for 24 h at 40 °C gave a significantly lower number of positive bottles compared with no delay (27.8 and 72.2% respectively, *P* = 0.018). When comparing the positive yields obtained between the different systems, a significant difference was detected between the two manual methods (*P* = 0.037).

### Time to positivity

**Fig. 1:**
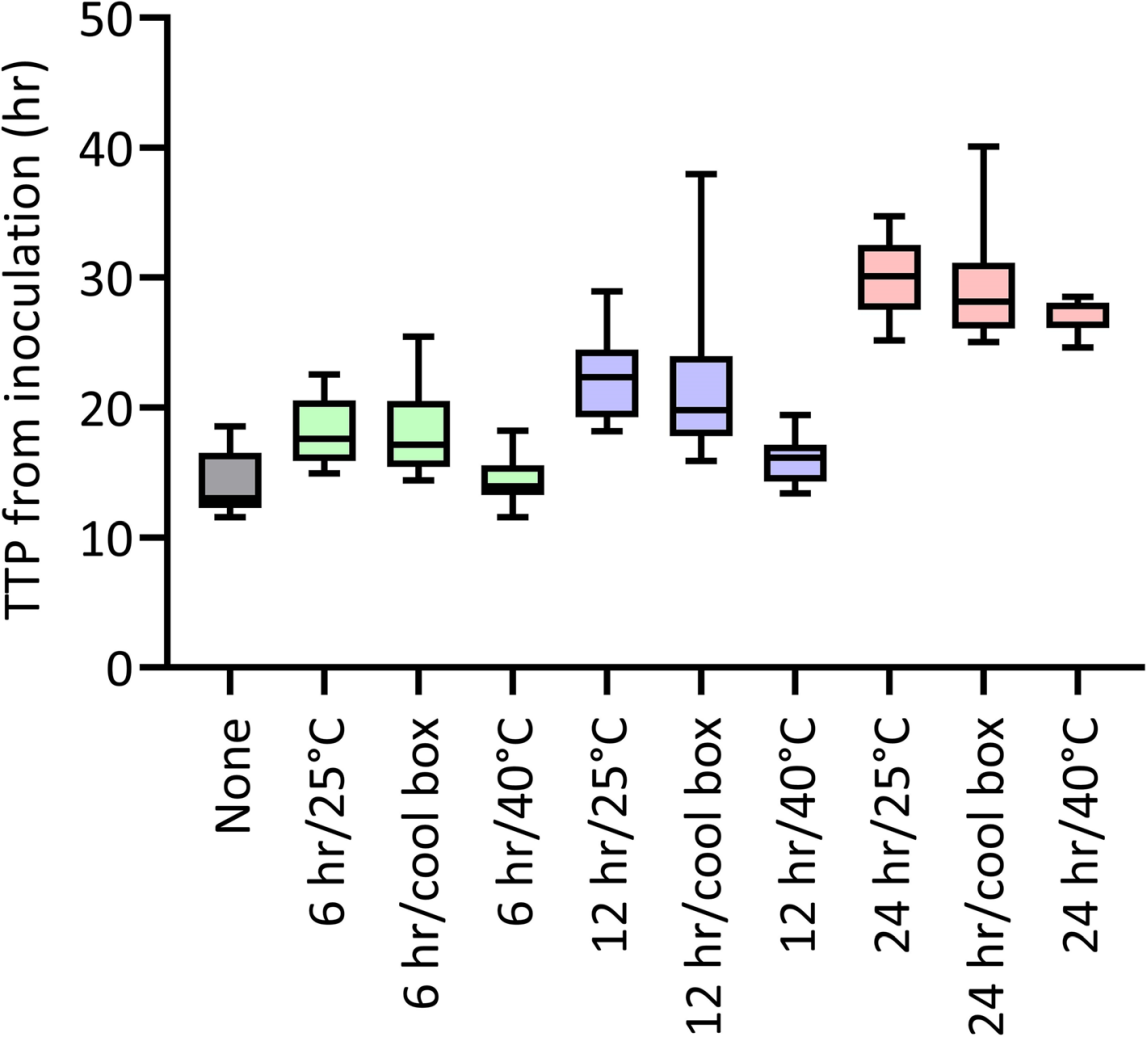
Time to positivity from time of inoculation for automated blood cultures. The box graph shows the time to positivity (TTP) for automated blood cultures for different storage conditions

The overall median TTP from inoculation for BCs performed with the automated systems was 18.9 h (IQR 15.6–26.0), with *E. coli* having the shortest TTP (15.9, IQR 14.2–25.2). Both *H. influenzae* (22.8, IQR 17.7–28.0) and *S. aureus* (22.1, IQR 17.6–28.0) had significantly longer TTP from inoculation than the other three bacterial species (*P* < 0.001–0.018). The overall median TTP increased from 13.0 h (IQR 12.3–16.4) with no delay, to 28.1 h (IQR 26.8–30.9) after 24 h of storage (*P* for trend < 0.001) (Fig. 1). There was no significant difference in the TTP between the two automated BC systems.

For manual BC systems the majority of positive sub-cultures was obtained for the first 24 h sub-culture (393/409, 96.1%) and no new positive sub-culture was obtained after 4 days of incubation. The 16 positive bottles that had positive sub-cultures following > 24 h incubation had been stored at 25 °C for 24 h (eight bottles); in a cool-box for 24 h (five bottles); in a cool-box for 12 h (two bottles) or at 25 °C for 6 h (1 bottle). Of the 16 bottles that had positive sub-cultures following > 24 h incubation, two had been cultured at LOMWRU and 14 had been cultured at COMRU (0.9 and 7.2% of positive bottles at these sites respectively, *P* = 0.001).

### Visual detection of growth

Detection of growth by visual inspection was recorded daily for manual methods (with the exception of *S. pneumoniae* at COMRU, which was omitted in error). There was discordance in both directions when comparing visual detection of growth with detection of growth obtained by sub-culturing, however, overall visual detection significantly underestimated the number of actual sub-culture positive bottles with 288/450 (64%) versus 362/450 (80.4%) determined to be positive respectively (*P* < 0.001).

Visual detection of growth prior to incubation is possible for BacT/ALERT® PF Plus bottles that have a colorimetric growth sensor. Recording of BacT/ALERT® bottle sensor colours showed growth before incubation increased with increased time to incubation (Table 2). Fewer bottles that indicated growth prior to incubation flagged positive (62/75, 82.7%) compared to those that did not indicate growth prior to incubation (164/175, 93.7%) (*P* = 0.01). Of the 13 bottles with positive growth indicators that failed to flag positive, 12 had been stored at 40 °C for ≥ 12 h.

## Discussion

There are many factors that can affect pathogen recovery from BCs, from the processes of blood collection through to the methodologies used in the laboratory. There are guidelines describing best practice, but these do not sufficiently address how to manage delayed incubation, particularly in tropical climates. A review describing the best practice for BCs in LMICs highlights the issue of delayed incubation, however, optimal conditions have not been established [9]. The UK Standard for Microbiology Investigations for BCs recommends that bottles should be placed in incubators no later than 4 h after inoculation [10]. This is not always possible; for example the laboratory in Laos receives BCs from > 300 km away, and only once per day. This study set out to determine the impact of such, sometimes unavoidable, delays on BC results.

### *S. pneumoniae* autolysis

In this study when using the BacT/Alert system, pneumococci were recovered from 11/11 of positive BC bottles sub-cultured < 7 h from the time they flagged positive and just 12/31 when sub-cultured after 7 h. The reduction in recovery was most likely due to autolysis, which has been described previously for the BacT/Alert automated system with similar recovery rates, and the continuous agitation speculated to contribute to the phenomenon [11–15]. This finding supports recommendations that positive BC bottles in automated systems should be sub-cultured immediately to minimize the risk of false negative results and reporting delays [16], with implications for laboratories that are not staffed 24 h/7 days a week, a common occurrence in LMIC settings.

### Impact of delayed incubation on yield

The main aim of this study was to assess the impact of delayed incubation on positive BC bottle yield. It is encouraging that in this study no significant differences in positivity compared with no delay were obtained when inoculated bottles were stored for 6 h or 12 h irrespective of the storage temperature. When storing bottles for 24 h the temperature was found to be important with the lowest yield of 65.6% obtained for bottles stored at 40 °C; compared with 78.9 and 91.1% when stored in a cool-box or at 25 °C respectively. Ambient temperature is usually recommended for transportation of BCs; however, the results presented here indicate that this may be suboptimal in tropical climates and bottles should be protected from high temperatures where possible. This agrees with a review on how to optimize BCs that stated that pre-incubation at 35 °C should be discouraged [17]. In addition, a previous study comparing delayed incubation at room temperature with 35 °C, found positive BC yields decreased with increasing delay time more in BCs stored at 35 °C compared with room temperature [18].

To provide a relatively protected environment at ambient temperatures, the use of cool-boxes without ice packs offers a cost-effective option, and promisingly the positivity rates in this study were higher for samples held in cool-boxes as opposed to 40 °C. Nevertheless, some caution must be used when taking this approach, for example during periods of very high ambient temperature or when exposure of the boxes to direct sunlight cannot be avoided. The highest recorded temperature inside the cool-boxes was 31.0 °C, but temperatures > 40 °C have been recorded previously [19] and were observed in this study when temperature monitoring continued (Table S2, Figures S1, S2 and S3). Further studies into the utility of cool-boxes in tropical climates with and without ice packs are warranted as this is a practicable method of storage and transportation for samples in LMICs.

### Impact of delayed incubation on time to positivity

Although delays before incubation did not affect the proportion of positive BCs if the temperature was controlled, they did affect the TTP from bottle inoculation. This was illustrated best by the automated systems where the median TTP increased from 13.0 h (IQR 12.3–16.4) with no delay to 28.1 h (IQR 26.8–30.9) following 24 h of storage (*P* for trend < 0.001) (Fig. 1). This finding agrees with a previous study that demonstrated TTP increased for samples kept at room temperature for 19 h compared with 2 h or 9.5 h (*P* < 0.001) [20]. This has implications for result reporting and patient management, with potential negative impacts on patient outcome if inadequate empiric treatment has been used. For example, a previous study found TTP was inversely associated with mortality risk for patients with Gram negative bacteremia [21]. This provides further evidence that delays to incubation should be avoided where possible.

### Visual detection of growth

When processing manual BCs, reliance on visual detection of growth is dependent on local standard operating procedures. The accuracy of visual detection of growth is multifactorial and may vary between individuals [9]. In this study visual detection of growth significantly underestimated the the number of positive bottles detected by sub-culture (*P* < 0.001), illustrating that visual detection of growth should not be relied upon.

Recording of BacT/ALERT® bottle sensor colours prior to incubation showed growth before incubation increased with increased time to incubation and that fewer bottles that indicated growth prior to incubation flagged positive compared to those that did not indicate growth prior to incubation (*P* = 0.01). Of the 13 bottles with positive growth indicators that failed to flag positive, 12 had been stored at 40 °C for ≥12 h. Failure of bottles to flag positive despite indicating growth prior to incubation may have been due to loss of viability and/or due to insufficient changes in the colorimetric sensor for the machine to detect (exhaustive CO_2_ production prior to incubation).

### Utility of blind sub-cultures

Results from this study including delayed TTP, failure of positive bottles to flag positive in the automated BC system and unreliability of visual checks for growth for the manual systems indicate that a blind sub-culture before incubation may help to reduce false negative results and decrease TTP from inoculation if BC bottles are unavoidably stored for extended periods of time. Previous studies evaluating the utility of blind sub-cultures for diagnostic BCs concluded that early blind sub-cultures were useful, with one study recommending performing a blind sub-culture 12–17 h following inoculation and one 4–14 h following arrival in the laboratory [22–24]. In practice, the utility, practicability and timing of an early blind sub-culture will depend on laboratory and biological factors including working hours, staffing levels, workload, blood volumes and bacterial loads. The majority of manual culture bottles were positive for their first sub-culture performed at 24 h (96%), further supporting the use of blind sub-cultures, with at least one being performed following 24 h of incubation. This is in agreement with previous studies and guidelines [10, 25].

### Automated versus manual blood cultures

In this study, both manual and automated systems were included; these reflected the systems in-use at the different sites at the time. Overall, the automated systems performed better than the manual systems resulting in significantly more positive BC bottles (356/399, 89.2% versus 409/500, 81% respectively; *P* = 0.002). In addition to other benefits, such as continuous-monitoring to enable positive cultures to be detected sooner and savings of staff time, automated systems have previously been reported to have increased sensitivity and decreased time to positivity compared with manual BCs [26]. This finding supports the use and implementation of automated BC bottles in all settings, where financially viable. Significant differences were detected when yield and TTP from inoculation were compared for the two manual BC systems that may have resulted from the different broths used, highlighting the advantage of standardized automated BC bottles.

### Study design and limitations

As previously shown, low-colony-count neonatal sepsis can occur [27, 28]. In this study a 2 ml inoculum containing approximately 3 CFU/ml was chosen to represent the lowest concentration suitable to conduct the experiments. Although the study was designed to ensure the results were generalizable, it did have some limitations. When stratified by organism, storage conditions and BC system, the sample sizes were small. Different healthy volunteers donated blood, opposed to infected individuals with unknown impact. The experiments were challenging to perform and for practical reasons a limited pathogen/strain range was used, with only one blood inoculum volume. In this study, the recovery of *Streptococcus* spp. were more susceptible to the adverse effects of storage. In reality, the pathogen range seen in routine practice is much greater, and organisms encountered, particulary as part of a natural infection, may be affected by different storage conditions to a lesser or greater extent than described here. In addition, the impact of delays to incubation on polymicrobial infections were not investigated. It is possible that the presence of contaminating organisms could overgrow true pathogens with longer delays to incubation.

Variation in results was observed between the different sites and for the different organisms. This was anticipated, however, the results for some organisms varied more than expected. For example, the positive yield of *E. coli* was just 36% (18/50) at COMRU compared with 97.7% (127/130) at other sites (Table S1). Differing inocula would not explain the variation seen for *E. coli* (range 2–3 CFU/ml), and the same batch of media used for the *E. coli* experiment supported the growth of more fastidious organisms. It is therefore probable that there was a technical issue for this experiment, for example inadequate mixing or an interaction between the bacteria and the donor blood.

## Conclusions

The results from this study, together with findings from other studies, lead to the following recommendations:

- BC storage time should be minimized where possible (preferably no longer than 12 h);
- if storage for longer than 12 h is unavoidable, BC bottles should be protected from high temperatures, for example by storing them in a cool-box (maintaining a temperature of approximately 25 °C was shown to be suitable in this study) away from direct sunlight;
- a blind sub-culture prior to incubation on all BC bottles that have had prolonged storage should be performed (preferably on all bottles that have been stored for ≥ 12 h);
- for manual BC systems, visual inspection alone should not be relied upon and at least one sub-culture following 24 h incubation should be performed; and
- for automated BC systems, delays in sub-culturing bottles that have flagged positive should be minimized (this and a previous study support a maximum delay of 7 h to maximize the recovery of *S. pneumoniae*).

This study provides useful insight into the impact of different storage conditions prior to incubation on BC results. Although improved access to microbiology services in LMICs is the aspiration, this study provides some reassurance that unavoidable delays in incubating BCs can be managed to minimize any negative impacts.

## Supplementary Information

**Additional file 1: Table S1.** Experiment details and numbers of positive blood culture bottles, irrelevant of storage condition, per organism. **Tables S2-S6.** Number of positive blood culture bottles for different storage conditions for each organism. **Tables S7.** Cool box temperatures during and following the study period. **Figure S1-S3.** Line graphs of cool box temperatures obtained during and following the study period for LOMWRU, COMRU and SMRU.

## Data Availability

The dataset supporting the conclusions of this article is available in the Oxford University Research Archive (ORA) repository (https://ora.ox.ac.uk/objects/uuid:8c023bb7-496c-4926-a556-f38d0c289c4e) (DOI: 10.5287/bodleian:NGO7xqboq).

## Abbreviations

COMRU: Cambodia Oxford Research Unit
LMICs: Low and middle-income countries
LOMWRU: Lao-Oxford-Mahosot Hospital-Wellcome Trust Research Unit
SMRU: Shoklo Malaria Research Unit
TTP: Time to positivity
