# Supplementary material for: Impact of delays to incubation and storage temperature on blood culture results: a multi-centre study
Source: BMC Infect Dis. 2021 Feb 12;21:173. doi: 10.1186/s12879-021-05872-8 (PMC7881545; doi:10.1186/s12879-021-05872-8)
Supplement: Supplementary file 1 — Additional file 1: Table S1. Experiment details and numbers of positive blood culture bottles, irrelevant of storage condition, per organism. Tables S2-S6. Number of positive blood culture bottles for different storage conditions for each organism. Tables S7. Cool box temperatures during and following the study period. Figure S1-S3. Line graphs of cool box temperatures obtained during and following the study period for LOMWRU, COMRU and SMRU. [file 12879_2021_5872_MOESM1_ESM.docx]

**Additional File 1**

**Supplemental Tables**

**Table S1:** Experiment details and numbers of positive blood culture bottles, irrespective of storage condition, per organism.

| **Site** | **System** | **Pathogen** | **CFU/ml** | **Volunteer** | **Cool box median temperature, °C (IQR)** | **No. positive bottles (%)^a^** |
| --- | --- | --- | --- | --- | --- | --- |
| COMRU | Manual | *Escherichia coli* | 2.0 | 3 | 29.3 (29.3-29.8) | 18/50 (36.0) |
| LOMWRU | Manual | *Escherichia coli* | 2.3 | 6 | 27.9 (26.3-29.8) | 50/50 (100) |
| LOMWRU | Automated | *Escherichia coli* | 2.3 | 6 | 27.9 (26.3-29.8) | 27/30 (90.0) |
| SMRU | Automated | *Escherichia coli* | 3.0 | 10 | 27.1 (26.9-27.3) | 50/50 (100) |
| COMRU | Manual | *Haemophilus influenzae* | 3.2 | 5 | 26.7 (26.1-27.5) | 41/50 (82.0) |
| LOMWRU | Manual | *Haemophilus influenzae* | 4.5 | 8 | 26.2 (24.6-28.2) | 50/50 (100) |
| LOMWRU | Automated | *Haemophilus influenzae* | 4.5 | 8 | 26.2 (24.6-28.2) | 30/30 (100) |
| SMRU | Automated | *Haemophilus influenzae* | 7.0 | 13 | 27.2 (26.9-27.5) | 50/50 (100) |
| COMRU | Manual | *Streptococcus agalactiae* | 2.3 | 1 | 27.0 (26.4-27.7) | 39/50 (78.0) |
| LOMWRU | Manual | *Streptococcus agalactiae* | 4.1 | 8 | 26.5 (24.6-29.3) | 50/50 (100) |
| LOMWRU | Automated | *Streptococcus agalactiae* | 4.1 | 8 | 26.5 (24.6-29.3) | 30/30 (100) |
| SMRU | Automated | *Streptococcus agalactiae* | 7.0 | 10 | 26.2 (26.0-26.5) | 43/50 (86.0) |
| COMRU | Manual | *Staphylococcus aureus* | 1.7 | 4 | 29.1 (28.6-29.2) | 50/50 (100) |
| LOMWRU | Manual | *Staphylococcus aureus* | 2.2 | 7 | 24.8 (20.9-26.7) | 47/50 (94.0) |
| LOMWRU | Automated | *Staphylococcus aureus* | 2.2 | 7 | 24.8 (20.9-26.7) | 30/30 (100) |
| SMRU | Automated | *Staphylococcus aureus* | 2.9 | 11 | 26.6 (26.4-27.2) | 41/50 (82.0) |
| COMRU | Manual | *Streptococcus pneumoniae* | 1.0 | 2 | 26.7 (25.8-27.0) | 47/50 (94.0) |
| LOMWRU | Manual | *Streptococcus pneumoniae* | 1.7 | 9 | 25.4 (22.2-27.8) | 17/50 (34.0) |
| LOMWRU | Automated | *Streptococcus pneumoniae* | 1.7 | 9 | 25.4 (22.2-27.8) | 13/29 (44.8)^b^ |
| SMRU | Automated | *Streptococcus pneumoniae* | 10.0 | 12 | 27.5 (27.2-27.7) | 42/50 (84.0) |

^a^For automated systems this is the number of bottles that flagged positive, and for manual systems this is the number of bottles that were positive on sub-culture; ^b^One bottle excluded due to contamination.

Table S2 Number of positive blood culture bottles inoculated with *E. coli* for different storage conditions.

| Storage condition | SMRU^a^ | LOMWRU^a^ | LOMWRU^b^ | COMRU^b^ | Total |
| --- | --- | --- | --- | --- | --- |
| No storage | 5/5 | 3/3 | 5/5 | 0/5 | 13/18 |
| 6 hr 25°C | 5/5 | 3/3 | 5/5 | 4/5 | 17/18 |
| 6 hr cool box | 5/5 | 3/3 | 5/5 | 3/5 | 16/18 |
| 6 hr 40°C | 5/5 | 3/3 | 5/5 | 0/5 | 13/18 |
| 12 hr 25°C | 5/5 | 3/3 | 5/5 | 1/5 | 14/18 |
| 12 hr cool box | 5/5 | 3/3 | 5/5 | 3/5 | 16/18 |
| 12 hr 40°C | 5/5 | 3/3 | 5/5 | 0/5 | 13/18 |
| 24 hr 25°C | 5/5 | 3/3 | 5/5 | 4/5 | 17/18 |
| 24 hr cool box | 5/5 | 3/3 | 5/5 | 3/5 | 16/18 |
| 24 hr 40°C | 5/5 | 0/3 | 5/5 | 0/5 | 10/18 |
| Total | 50/50 (100%) | 27/30 (90%) | 50/50 (100%) | 18/50 (36%) | 145/180 (81%) |

^a^ = automated system (positive bottles are those that flagged positive in the machine)

^b^ = manual system (positive bottles are those that were positive on sub-culture)

Table S3 Number of positive blood culture bottles inoculated with *H. influenzae* for different storage conditions.

| Storage condition | SMRU^a^ | LOMWRU^a^ | LOMWRU^b^ | COMRU^b^ | Total |
| --- | --- | --- | --- | --- | --- |
| No storage | 5/5 | 3/3 | 5/5 | 5/5 | 18/18 |
| 6 hr 25°C | 5/5 | 3/3 | 5/5 | 5/5 | 18/18 |
| 6 hr cool box | 5/5 | 3/3 | 5/5 | 4/5 | 17/18 |
| 6 hr 40°C | 5/5 | 3/3 | 5/5 | 4/5 | 17/18 |
| 12 hr 25°C | 5/5 | 3/3 | 5/5 | 2/5 | 15/18 |
| 12 hr cool box | 5/5 | 3/3 | 5/5 | 4/5 | 17/18 |
| 12 hr 40°C | 5/5 | 3/3 | 5/5 | 5/5 | 18/18 |
| 24 hr 25°C | 5/5 | 3/3 | 5/5 | 5/5 | 18/18 |
| 24 hr cool box | 5/5 | 3/3 | 5/5 | 3/5 | 16/18 |
| 24 hr 40°C | 5/5 | 3/3 | 5/5 | 4/5 | 17/18 |
| Total | 50/50 (100%) | 30/30 (100%) | 50/50 (100%) | 41/50 (82%) | 171/180 (95%) |

^a^ = automated system (positive bottles are those that flagged positive in the machine)

^b^ = manual system (positive bottles are those that were positive on sub-culture)

Table S4 Number of positive blood culture bottles inoculated with *S. agalactiae* for different storage conditions.

| Storage condition | SMRU^a^ | LOMWRU^a^ | LOMWRU^b^ | COMRU^b^ | Total |
| --- | --- | --- | --- | --- | --- |
| No storage | 5/5 | 3/3 | 5/5 | 5/5 | 18/18 |
| 6 hr 25°C | 5/5 | 3/3 | 5/5 | 4/5 | 17/18 |
| 6 hr cool box | 5/5 | 3/3 | 5/5 | 4/5 | 17/18 |
| 6 hr 40°C | 5/5 | 3/3 | 5/5 | 4/5 | 17/18 |
| 12 hr 25°C | 5/5 | 3/3 | 5/5 | 4/5 | 17/18 |
| 12 hr cool box | 5/5 | 3/3 | 5/5 | 5/5 | 18/18 |
| 12 hr 40°C | 5/5 | 3/3 | 5/5 | 5/5 | 18/18 |
| 24 hr 25°C | 5/5 | 3/3 | 5/5 | 5/5 | 18/18 |
| 24 hr cool box | 2/5 | 3/3 | 5/5 | 0/5 | 10/18 |
| 24 hr 40°C | 1/5 | 3/3 | 5/5 | 3/5 | 12/18 |
| Total | 43/50 (86%) | 30/30 (100%) | 50/50 (100%) | 39/50 (78%) | 162/180 (90%) |

^a^ = automated system (positive bottles are those that flagged positive in the machine)

^b^ = manual system (positive bottles are those that were positive on sub-culture)

Table S5 Number of positive blood culture bottles inoculated with *S. aureus* for different storage conditions.

| Storage condition | SMRU^a^ | LOMWRU^a^ | LOMWRU^b^ | COMRU^b^ | Total |
| --- | --- | --- | --- | --- | --- |
| No storage | 5/5 | 3/3 | 5/5 | 5/5 | 18/18 |
| 6 hr 25°C | 4/5 | 3/3 | 5/5 | 5/5 | 17/18 |
| 6 hr cool box | 5/5 | 3/3 | 5/5 | 5/5 | 18/18 |
| 6 hr 40°C | 3/5 | 3/3 | 5/5 | 5/5 | 16/18 |
| 12 hr 25°C | 4/5 | 3/3 | 5/5 | 5/5 | 17/18 |
| 12 hr cool box | 5/5 | 3/3 | 5/5 | 5/5 | 18/18 |
| 12 hr 40°C | 2/5 | 3/3 | 5/5 | 5/5 | 15/18 |
| 24 hr 25°C | 5/5 | 3/3 | 5/5 | 5/5 | 18/18 |
| 24 hr cool box | 4/5 | 3/3 | 4/5 | 5/5 | 16/18 |
| 24 hr 40°C | 4/5 | 3/3 | 3/5 | 5/5 | 15/18 |
| Total | 41/50 (82%) | 30/30 (100%) | 47/50 (94%) | 50/50 (100%) | 168/180 (93%) |

^a^ = automated system (positive bottles are those that flagged positive in the machine)

^b^ = manual system (positive bottles are those that were positive on sub-culture)

Table S6 Number of positive blood culture bottles inoculated with *S. pneumoniae* for different storage conditions.

| Storage condition | SMRU^a^ | LOMWRU^a^ | LOMWRU^b^ | COMRU^b^ | Total |
| --- | --- | --- | --- | --- | --- |
| No storage | 5/5 | 1/3 | 2/5 | 5/5 | 13/18 |
| 6 hr 25°C | 4/5 | 3/3 | 4/5 | 5/5 | 16/18 |
| 6 hr cool box | 5/5 | 3/3 | 0/5 | 5/5 | 13/18 |
| 6 hr 40°C | 5/5 | 0/3 | 1/5 | 5/5 | 11/18 |
| 12 hr 25°C | 4/5 | 1/3 | 4/5 | 4/5 | 13/18 |
| 12 hr cool box | 4/5 | 0/2^c^ | 2/5 | 5/5 | 11/17 |
| 12 hr 40°C | 5/5 | 2/3 | 1/5 | 5/5 | 13/18 |
| 24 hr 25°C | 5/5 | 2/3 | 1/5 | 3/5 | 11/18 |
| 24 hr cool box | 5/5 | 1/3 | 2/5 | 5/5 | 13/18 |
| 24 hr 40°C | 0/5 | 0/3 | 0/5 | 5/5 | 5/18 |
| Total | 42/50 (84%) | 13/29 (45%) | 17/50 (34%) | 47/50 (94%) | 119/179 (66%) |

^a^ = automated system (positive bottles are those that flagged positive in the machine)

^b^ = manual system (positive bottles are those that were positive on sub-culture)

^c^ = one bottle excluded due to contamination

**Table S7:** Cool box temperatures during and following the study period.

| Site | Months | Days | Min (°C) | Max (°C) | Median (°C) | IQR (°C) |
| --- | --- | --- | --- | --- | --- | --- |
| LOMWRU | November to May | 167 | 16.6 | 41.3 | 27.0 | 24.4 – 30.3 |
| COMRU | November to April | 156 | 24.3 | 32.5 | 28.8 | 27.9 – 29.6 |
| SMRU | October to March^a^ | 140 | 21.4 | 30.9 | 26.8 | 26.1 – 27.3 |
| All | / | / | 16.6 | 41.3 | 27.6 | 26.3 – 29.2 |

In total, 96 readings per day / per site were analysed. ^a^No readings were available for 8 days during this period.

**Supplemental Figures**

**Figure S1:** Line graph of cool box temperatures obtained during and following the study period for LOMWRU.

A total of 96 temperature readings were plotted per day.

**Figure S2:** Line graph of cool box temperatures obtained during and following the study period for COMRU.

A total of 96 temperature readings were plotted per day.

**Figure S3:** Line graph of cool box temperatures obtained during and following the study period for SMRU.

A total of 96 temperature readings were plotted per day. No readings were available for 8 days in February.
